# Residual Stress Distribution in Dievar Tool Steel Bars Produced by Conventional Additive Manufacturing and Rotary Swaging Processes

**DOI:** 10.3390/ma17235706

**Published:** 2024-11-22

**Authors:** Josef Izák, Pavel Strunz, Olena Levytska, Gergely Németh, Jan Šaroun, Radim Kocich, Marek Pagáč, Kostyantyn Tuharin

**Affiliations:** 1 Faculty of Mechanical Engineering, Brno University of Technology, Technická 2896, 616 00 Brno, Czech Republic; 183119@vutbr.cz; 2 Nuclear Physics Institute of the Czech Academy of Sciences, Husinec—Řež 130, 250 68 Řež, Czech Republicnemeth@ujf.cas.cz (G.N.); saroun@ujf.cas.cz (J.Š.);; 3 Paul Scherrer Institute—PSI, 5232 Villigen, Switzerland; 4 Faculty of Materials Science and Technology, VŠB-Technical University of Ostrava, 17. Listopadu 2172/15, 708 00 Ostrava, Czech Republic; radim.kocich@vsb.cz; 5 Faculty of Mechanical Engineering, VŠB-Technical University of Ostrava, 17. Listopadu 2172/15, 708 00 Ostrava, Czech Republic; 6 Faculty of Mathematics and Physics, Charles University, Ke Karlovu 2027/3, 121 16 Prague, Czech Republic

**Keywords:** neutron diffraction, Dievar, tool steel, hot work tool steel, additive manufacturing, SLM, selective laser melting, rotary swaging, residual stress

## Abstract

The impact of manufacturing strategies on the development of residual stresses in Dievar steel is presented. Two fabrication methods were investigated: conventional ingot casting and selective laser melting as an additive manufacturing process. Subsequently, plastic deformation in the form of hot rotary swaging at 900 °C was applied. Residual stresses were measured using neutron diffraction. Microstructural and phase analysis, precipitate characterization, and hardness measurement—carried out to complement the investigation—showed the microstructure improvement by rotary swaging. The study reveals that the manufacturing method has a significant effect on the distribution of residual stresses in the bars. The results showed that conventional ingot casting resulted in low levels of residual stresses (up to ±200 MPa), with an increase in hardness after rotary swaging from 172 HV1 to 613 HV1. SLM-manufactured bars developed tensile hoop and axial residual stresses in the vicinity of the surface and large compressive axial stresses (−600 MPa) in the core due to rapid cooling. The subsequent thermomechanical treatment via rotary swaging effectively reduced both the surface tensile (to approximately +200 MPa) and the core compressive residual stresses (to −300 MPa). Moreover, it resulted in a predominantly hydrostatic stress character and a reduction in von Mises stresses, offering relatively favorable residual stress characteristics and, therefore, a reduction in the risk of material failure. In addition to the significantly improved stress profile, rotary swaging contributed to a fine grain (3–5 µm instead of 10–15 µm for the conventional sample) and increased the hardness of the SLM samples from 560 HV1 to 606 HV1. These insights confirm the utility of rotary swaging as a post-processing technique that not only reduces residual stresses but also improves the microstructural and mechanical properties of additively manufactured components.

## 1. Introduction

The mechanical properties and service life of components made from tool steels are inherently influenced by the presence, distribution, and magnitude of residual stresses within the material [1,2,3]. Residual stresses, which are self-equilibrating internal stresses that remain in a material after all external forces have been removed [4], are a critical factor in determining the mechanical behavior of these materials in various industrial applications such as aerospace, automotive, structural components, railway transport, etc. [5,6,7,8,9]. These stresses can arise from manufacturing processes [10], phase transformations [11], or thermal cycling [10], and, depending on their nature and distribution, can be either beneficial or detrimental. Residual stresses can lead to a variety of undesirable consequences such as dimensional instability, distortion, fatigue failure under cyclic loading conditions [3,12,13], or in last stadium in initiation, propagation crack and delamination [14,15,16,17]. This only happens if the residual stresses in the material reach the yield stress and damage to the component occurs [14,18]. On the other hand, compressive residual stresses, particularly those located on the surface of a component, can have a beneficial effect by improving fatigue resistance and delaying crack initiation [19,20].

Current research has extensively explored residual stresses in conventionally manufactured tool steels; however, studies addressing these phenomena in additively manufactured tool steels, particularly under mechanical post-processing, remain scarce. This knowledge gap hinders the full utilization of advanced manufacturing techniques in industries demanding high-performance components. This study aims to address this gap, providing insights into the unique interplay between additive manufacturing techniques and mechanical treatments.

In the context of tool steels, residual stresses can significantly affect properties such as fatigue strength [12], wear resistance [21,22], dimensional stability [23], and overall mechanical performance [24,25]. Tool steels, particularly those used in high-temperature applications, are frequently exposed to severe thermal and mechanical stresses, necessitating a detailed understanding of residual stress evolution in these materials to enhance their performance and reliability. One such tool steel that has gained prominence for use in high-temperature applications such as die casting and hot forging is Dievar [26]. Dievar is a chromium–molybdenum–vanadium alloy hot work tool steel renowned for its excellent combination of toughness, thermal fatigue resistance, and high temperature strength [27,28]. However, despite these advantages, Dievar—like many other high alloy tool steels—is subject to the development of significant residual stresses due to its high hardenability and the complex phase transformations [11] that occur during manufacturing processes, particularly heat treatment.

Traditional manufacturing processes for tool steels involve casting, extensive forging or rolling, and a series of heat treatments such as annealing, hardening, and tempering. Each of these processes can lead to residual stresses that must be carefully managed to maintain the structural integrity of the component. Soft annealing is a heat treatment used to improve the machinability of tool steels and prepare their microstructure for subsequent hardening. It is typically carried out at temperatures where carbides dissolve and a uniform microstructure can be achieved. Slow cooling after annealing is intended to reduce residual stresses by promoting uniform thermal contraction. On the other hand, the hardening process, which typically starts with austenitization followed by quenching, introduces significant residual stresses due to the rapid cooling and subsequent temperature gradients [29,30,31]. The transformation from austenite, which has a face-centered cubic (FCC) crystal structure, to martensite, which changes to a body-centered tetragonal (BCT) structure, is particularly critical [5]. The martensitic transformation, driven by a critical cooling rate, is accompanied by a volume expansion that generates residual tensile stresses at the surface and compressive stresses in the core of the component [32]. This transformation is often anisotropic as carbon atoms are unable to fully diffuse during rapid cooling, causing certain crystal axes to elongate, and contributing to the build-up of internal stresses. Subsequent tempering tends to reduce these stresses by transforming the martensite into more stable phases and relieving some of the internal stresses caused by quenching [33,34]. However, the challenge of controlling and modifying residual stress profiles in tool steels remains a significant problem even in conventional manufacturing.

Additive manufacturing (AM) has emerged as a transformative approach to produce complex geometries and customized parts that would be difficult or impossible to achieve using conventional manufacturing techniques [35,36,37]. A number of additive metal printing technologies exist, such as DED [38], PBF [39], and WAAM [40]. Among the various AM methods, selective laser melting (SLM) has gained particular attention for its ability to produce high-performance metal components with fine microstructures [41,42]. SLM, a kind of powder bed fusion process, involves a rapid melting of metal powders using a high-power laser, followed by rapid cooling and solidification. This process introduces steep thermal gradients and high cooling rates, resulting in a significant residual stress in the final component [43,44,45,46]. In many ways, the thermal cycling of materials during SLM is analogous to the quenching of large tool steel ingots, but on a much smaller scale. The structure after the SLM process is formed by dendrites in the build direction (i.e., heat dissipation) [47]. The high cooling rates achieved by SLM promote the formation of martensitic microstructures which, while beneficial for the mechanical strength, can also lead to high levels of internal stress [3]. The resulting martensitic structure often requires no additional post heat treatment as the rapid cooling inherent in the process naturally produces a fine-grained martensite [48,49,50,51]. However, the high residual tensile stresses introduced by the SLM process can affect the mechanical performance and dimensional stability of the final component. The removal of these stresses, often through stress-relieving treatments such as annealing, is therefore critical for ensuring the structural integrity of SLM-manufactured components [52,53,54]. Last but not least, printing parameters such as the layer thickness [55], the scan speed, the laser power, the scan pattern, the hatch spacing, the component orientation, etc., have a major influence on the amount of internal stress [13,56,57,58,59,60].

An alternative approach to varying the residual stresses, particularly in tool steels and other high-strength alloys, is the use of mechanical post-processing techniques such as rotary swaging [61,62,63]. Rotary swaging is a cold or hot forming process in which the diameter of bars or tubes is reduced by radial deformation, typically using a series of rotating dies. Unlike heat treatments, which relieve residual stresses by promoting microstructural changes through thermal energy, rotary swaging achieves residual stress modification through mechanical deformation. The process refines the microstructure of the material by inducing plastic deformation and—at the same time—can introduce beneficial residual compressive stresses at the surface of the component. These compressive stresses are highly favorable in improving fatigue resistance and wear performance. In addition, the process has the advantage of grain refinement, which is associated with improved mechanical properties according to the Hall–Petch relationship [64,65]. For additive manufactured components, rotary swaging offers a potential solution to reducing the high residual tensile stresses that are typically introduced during rapid thermal cycling. By applying deformation, rotary swaging can reduce these stresses while refining the microstructure, thereby improving both the performance and the reliability of SLM-produced parts.

While conventional methods provide partial solutions, exploring advanced post-processing techniques such as rotary swaging opens new ways for residual stress management, particularly in additive manufacturing contexts. By integrating additive manufacturing with advanced mechanical post-processing, this research bridges material science, mechanical engineering, and manufacturing technology, paving the way for next-generation high-performance components.

One of the most effective techniques for determining residual stresses in materials, particularly those with complex geometries or thick sections, is neutron diffraction [66,67,68,69]. Neutron diffraction is a non-destructive method that allows the measurement of internal residual stresses deep within a material, providing a detailed map of stress distributions throughout the volume of a component. Unlike X-ray diffraction, which is limited to surface measurements [38,39], neutron diffraction can penetrate several centimeters into metallic components and measure three orthogonal strain components, making it an ideal tool for analyzing thick tool steel bars or additively manufactured parts [40].

Plenty of publications dealing with the non-destructive measurement of residual stresses in various materials by X-ray and ND exist. Examples of various materials include the following: Inconel 718 Ni-alloy [70], Ti-6Al-4V Ti-alloy [14], AISI 316L stainless steel [14,18], AlSi10Mg Al-alloy [71], etc. However, there are not many publications dealing with the non-destructive measurement of residual stresses in additively manufactured tool steels. Yan et al. discussed the microstructure and residual stresses in the AISI H13 tool steel. They found that the residual stress in the printed H13 material is in the range of 940–1420 MPa, which is nearing the yield strength of about 1650 MPa. The high residual stresses were mainly attributed to the martensitic transformation that occurred during SLM [41]. Yan et al. discussed the effects of both scanning patterns and cross-section geometries on the residual stresses and hardness [67].

The positive effects of plastic deformation using rotary swaging on refining the microstructures and thus affecting residual stress have been studied by many authors. Kunčická et al., Canelo-Yubero et al., and Strunz et al. investigated the effect of rotary swaging on residual stresses in the W-Ni-Co tungsten heavy alloy [62,63,72,73]. Canelo-Yubero et al. studied the residual stress distribution in a Cu/Al multifilament composite fabricated by rotary swaging [74]. Kunčická et al. investigated the analysis of deformation behavior and residual stress in rotary swaged Cu/Al clad composite wires, at temperatures of 20 °C and 250 °C [64], the effect of structure on residual stress and mechanical and electric properties in Cu/Al rotary swaged laminated Cu/Al composites [75], and sub-structure and residual stress in rotary swaged Cu/Al clad composite wires [76]. Ishkina et al. evaluated the effect of residual stress in rotary swaged E355 steel tubes [77,78]. Yuan et al. discussed the effect of annealing temperature on the texture and residual stress of Ti-6Al-4Valloy seamless tubing processed by cold rotary swaging [79]. Singh et al. conducted FEM analysis and an experimental evaluation of the residual stress of Zr-4 alloys processed using rotary swaging [80]. As far as the authors’ knowledge reaches, no such studies discussing the effects of rotary swaging on the structures and residual stress of tool steels have been published.

This study aims to investigate residual stress distributions in Dievar tool steel bars produced by conventional SLM additive manufacturing and rotary swaging processes. The findings of this study not only enhance the understanding of residual stress management in Dievar tool steel but also provide actionable insights for improving fatigue life and structural integrity in critical applications such as aerospace and automotive industries. By addressing a critical gap in the literature, this work facilitates a broader adoption of additive manufacturing combined with mechanical post-processing, helping to develop the next generation components for demanding applications.

## 2. Materials and Methods

### 2.1. Material and Samples’ Preparation

The investigated material was Dievar tool steel (Böhler-Uddeholm, Vienna, Austria). Its chemical composition can be seen in Table 1.

**Figure 1:**
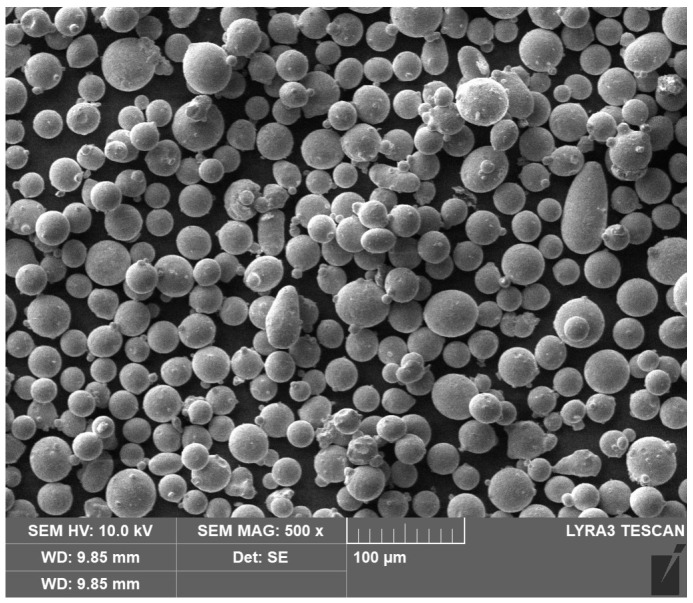
Powder used for SLM workpieces.

The basic material for the experiment was obtained by two approaches: (i) the conventional way using ingot melt casting (denoted as Conv in what follows) and (ii) additive manufacturing by selective laser melting (SLM). The Conv state was soft annealed at a temperature of 850 °C for two hours and cooled to 600 °C with a cooling rate of 10 °C/s, and then allowed to cool freely in air. The diameter of the resulting initial bar-shaped sample was 40.8 mm. A powder with an average size of 35 µm (Figure 1) was used for the second approach, i.e., SLM preparation of the initial samples. The material for the experiment was prepared using a Renishaw AM400 3D printer (By Renishaw plc, Wooton-under-Edge, UK). The powder was melted with a laser with a power of 165 W in an inert argon atmosphere. The parameters used for the 3D printing of samples were the thickness of the layer equal to 60 µm, point distance of 20 µm, hatch space of 0.110 µm, and exposure time of 54 µs. A chessboard pattern was used as a print strategy. Three bar samples were printed: rectangular with the cross-section of 12 × 12 mm^2^, cylindrical with the diameter of 12 mm, and cylindrical with the diameter of 25.4 mm. The last one was used as a workpiece for the subsequent rotary swaging process. The orientation of the workpiece during fabrication (printing) was 90°, i.e., the axis of the bar was perpendicular to the substrate. For additively manufactured samples with a diameter of 12 mm and with the rectangular cross-section of 12 × 12 mm^2^, the porosity did not exceed 33 µm. In the sample with a diameter of 25.4 mm, the porosity before rotary swaging was 13%. After rotary swaging, the porosity was reduced to 0.4%.

**Figure 2:**
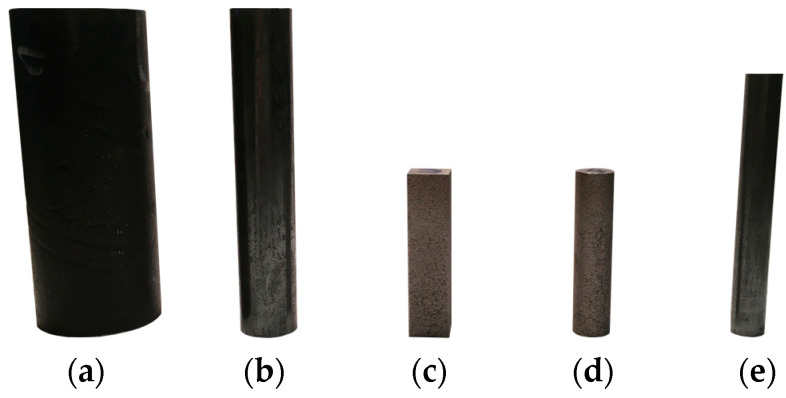
Workpieces’ measurement by neutron diffraction: (**a**) Conv, ø 40.8 mm; (**b**) Conv+RS, ø 18.8 mm; (**c**) SLM, 12 × 12 mm^2^; (**d**) SLM, ø 12 mm; (**e**) SLM + RS, ø 11.1 mm.

A multi-pass deformation using rotary swaging (RS) was applied afterwards at a temperature of 900 °C to both the Conv and SLM initial workpieces. The Conv workpiece was swaged from a diameter of 40.8 mm to 18.8 mm, and the SLM workpiece was swaged from a diameter of 25.4 mm to 11.1 mm. The swaging ratios after each swaging pass were calculated via the following relation:

(1) $$\varphi =\ln\left(\frac{S_{i}}{S_{n}}\right),$$

where *S_i_* and *S_n_* represent the cross-section areas at the input and output of the swaging dies, respectively. The swaging was carried out in five individual swaging passes and the swaging ratio for the Conv state was *φ*_Conv_ = 1.55 (Conv + RS) and for the SLM state *φ*_SLM_ = 1.65 (SLM + RS). An overview of the investigated samples can be seen in Table 2. A photo of the workpieces’ set can be seen in Figure 2.

### 2.2. Microstructure, Precipitates, and Hardness

The microstructures of the samples, phase composition, and precipitate arrangement were observed by a Zeiss EVO LS25 (TESCAN, Brno, Czech Republic) electron microscope available at Swansea University, and by TESCAN FERA3 GM (TESCAN, Brno, Czech Republic) scanning electron microscopy (SEM) equipment available at FZU, Institute of Physics of the CAS. Since the additive manufactured samples were printed with the same printing parameters, the microstructure, phase composition, precipitate analysis, and hardness were the same. Therefore, the SLM-circular sample (denoted as SLM in what follows) was used for the individual analysis evaluation. The samples were mechanically ground on SiC papers (Metalco s.r.o., Roztoky u Prahy, Czech Republic) and finally polished. Nital’s etchant (Metalco s.r.o., Roztoky u Prahy, Czech Republic) was used for the visualization of microstructure using SEM microscopy. The microstructure analysis was focused on observing the differences between the prepared states, i.e., Conv, Conv + RS, SLM, and SLM + RS.

The hardness was measured with the load of 1000 g (HV 1) using a Qness Q10A machine (Metalco Testing s.r.o., Roztoky u Prahy, Czech Republic). The microhardness was measured perpendicularly in section on all four samples, i.e., Conv, Conv + RS, SLM, and SLM + RS.

### 2.3. Neutron Diffraction

Neutron diffraction was used for the determination of elastic residual stresses in the above described conventionally prepared as well as SLM-fabricated Dievar steel samples. The aim was (i) to compare the residual stresses resulting from both preparation techniques, (ii) to compare the residual stresses in dependence on sample geometry (the cross-section of the SLM-circular and SLM-rectangular samples) to determine the suitable choice of the initial condition for the RS, and (iii) to assess the effect of rotary swaging on the residual stress state.

Due to the large penetration depth of the thermal neutrons in most materials, neutron diffraction is a suitable tool for the non-destructive measurement of residual elastic strains (followed by evaluation of stresses) within the bulk of large polycrystalline specimens [68].

The elastic strain measurement using neutron diffraction is based on the determination of the *d_hkl_* interplanar distance for the selected crystallographic plane (for the particularly oriented crystallites) from the measured angular position 2*θ_hkl_* of the *hkl* reflection. The *d_hkl_* spacing can be obtained using the Bragg law:

(2) $$\lambda =2d_{hkl}\;\sin\theta_{hkl},$$

where *λ* is the used neutron wavelength.

When the stress-free lattice spacing *d*_0,*hkl*_ is known, the lattice strain *ε^hkl^* can be calculated according to the following equation [68]:

(3) $$\varepsilon^{hkl}=\frac{d_{hkl}-d_{0,hkl}}{d_{0,hkl}}=\frac{\Delta d_{hkl}}{d_{0,hkl}}.$$

The strains evaluated in this way are elastic strains averaged over the gauge volume. Moreover, they are determined selectively only from those grains of the specimen which are suitably oriented.

One component of the elastic strain tensor can be evaluated for a single specimen orientation with respect to the scattering vector. For the other components, the other—mutually perpendicular in a standard case—sample orientations have to be used. In standard cases, the measurement of the three principal strain components $\varepsilon_{x}^{hkl}$, $\varepsilon_{y}^{hkl}$, $\varepsilon_{z}^{hkl}$ is carried out by a proper setting of three perpendiculatr orientations of the sample. These components are usually called the radial, hoop, and axial component for cylindric samples.

As can be seen from the above description, the measured parameters in the diffraction experiment are the lattice strain components in the gauge volume. For the determination of the stress tensor components, the generalized Hooke’s law is to be applied. For diffraction experiments, the formula for $\sigma_{x}$ stress component determination can be written in the following form [68]:

(4) $$\sigma_{x}=\frac{E_{hkl}}{1+\nu_{hkl}}\varepsilon_{x}^{hkl}+\frac{\nu_{hkl}\;E_{hkl}}{\left(1+\nu_{hkl}\right)\left(1-2\nu_{hkl}\right)}\left(\varepsilon_{x}^{hkl}+\varepsilon_{y}^{hkl}+\varepsilon_{z}^{hkl}\right).$$

where $\varepsilon_{x}^{hkl}$, $\varepsilon_{y}^{hkl}$, $\varepsilon_{z}^{hkl}$ are the *x*, *y*, *z* components of the lattice strain measured for the *hkl* crystal lattice plane, and *E_hkl_* and *ν_hkl_* are the diffraction elastic Young modulus and diffraction Poisson ratio, respectively [68]. The corresponding relations for the other stress components $\sigma_{y}$ and $\sigma_{z}$ are obtained by simple permutations of the *x*, *y*, and *z* indices.

The residual lattice strain in the provided sample was determined by neutron diffraction using the SPN-100 neutron diffractometer of the CANAM NPL infrastructure [81] (NPI CAS Řež, Husinec, Czech Republic), installed at the LVR-15 research reactor neutron source in Řež, CZ. The schematic sketch of the instrument can be found elsewhere [82]. This experimental facility is dedicated to the mapping of bulk-averaged residual strains in polycrystalline materials. The SPN-100 instrument is equipped with a horizontally bent Si monochromator (curvature 0.165 m^−1^), a radial collimator, and a position-sensitive detector for the fast recording of diffraction patterns. Sample positioning and its orientation to three mutually orthogonal orientations with respect to the scattering vector is carried out by means of a robotic arm.

The neutron wavelength of λ = 0.213 nm and the 110 reflection (2*θ*_110_ ≈ 63.4°) of the ferritic/martensitic phase were selected for the experiment. In some cases, the 111 reflection of the residual austenite was also observed. However, as this reflection was not visible in all the samples and for all the orientations, it was not used for stress evaluation.

The incident beam was formed by a Cd-mask (slit) of the width of 3 mm, and height between 4 and 30 mm. The large height was used for the hoop and radial strain components’ determination, where no change of the stress state along the cylindrical sample axis can be expected. The slit was positioned at 76 mm in front of the gauge volume center used for scanning the sample.

An oscillating radial collimator of 2 mm effective width was used for the diffracted beam determination for all the strain components. The slit and the radial collimator define the gauge volume. The 2*θ* angle position determined in the diffraction experiment was thus averaged over the elongated instrumental gauge volume with the cross-section of ≈6.5 mm^2^ and height of 4 ÷ 30 mm.

**Figure 3:**
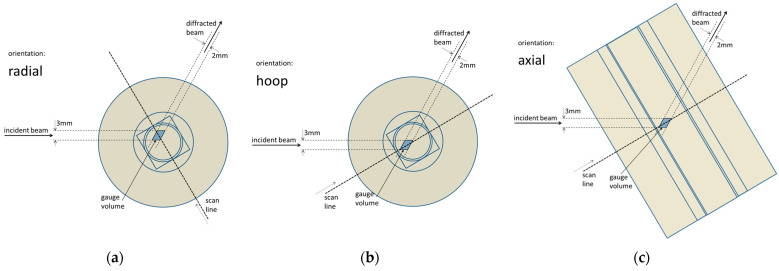
Scheme of the scanned sample with the elements of the measurement geometry for all three scanned components of the strain: (**a**) radial = *y*-component, (**b**) hoop = *x*-component, and (**c**) axial = *z*-component. The contours inside and at the edge of the color areas indicate the edges of the imaginary cross-sections of the individual five samples used for the investigation (see Table 2 for sample dimensions).

The sample was scanned through the gauge volume, always perpendicularly to the sample bar axis. The same scan line was used for all three strain components’ determination. The line went through the sample center. The positioning was conducted with high accuracy by using the robotic arm. The scan was performed with either a 0.5 or 1 mm increment, depending on the sample size and the gauge volume position near the surface or deeply in the bulk of the bar. The gauge volume, the line along which the scans were carried out, as well as the three sample orientations are indicated in Figure 3.

Only one strain component is determined for a single orientation of the sample—the one parallel to the scattering vector. The radial (*y*-component) characterizes the strain in the same direction as the scan line, the hoop (*x*-component) characterizes it in the direction perpendicular to the scan line and to the sample axis, and the axial (*z*-component) characterizes the strain in the direction perpendicular to the scan line and parallel to the sample axis.

The measuring time varied from 10 min to 2 h per point, depending on the texture, beam attenuation along the path inside the particular sample, and gauge volume height.

For the AM samples, the sample was also scanned along a second line, perpendicular to the first scan line and to the sample axis.

## 3. Results

### 3.1. Microstructure, Precipitates, and Hardness Evaluation

**Figure 4:**
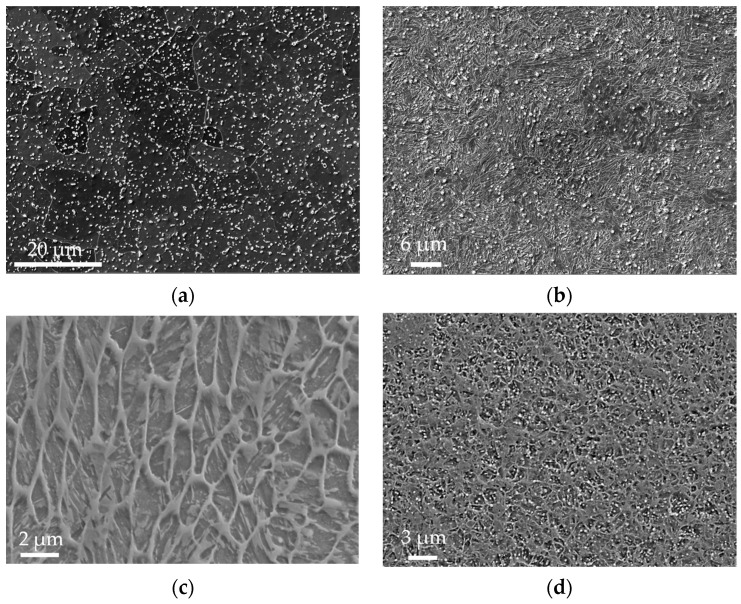
Microstructure of (**a**) Conv sample, mag. 7000×; (**b**) Conv + RS sample, 8000×; (**c**) SLM sample, mag. 6000×; (**d**) SLM + RS sample, 3000×.

**Figure 5:**
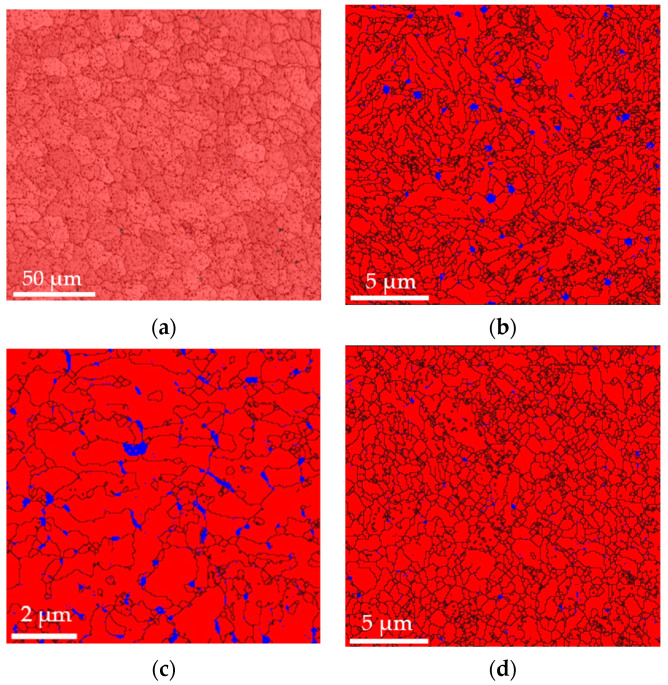
Proportion of BC(C,T)/FCC (red/blue) phase: (**a**) Conv sample, grid 200 × 200 µm^2^, step size 0.4 µm; (**b**) Conv+RS sample, grid 200 × 200 µm^2^, step size 0.1 µm; (**c**) SLM sample, grid 200 × 200 µm^2^, step size 0.05 µm; (**d**) SLM + RS sample, grid 200 × 200 µm^2^, step size 0.1 µm.

The microstructure of each individual state can be seen in Figure 4a–d. The phase composition is depicted in Figure 5. The local chemical compositions determined in several precipitates are shown in Figure 5a–d. The high local concentrations of Cr and Mo observed in certain areas indicate the presence of microstructural heterogeneities that arise due to the precipitation process during cooling. These phenomena are typical under cooling conditions, leading to the formation of secondary phases enriched in Cr and Mo. While the initial metal powder contains only 5% Cr and 2% Mo, the local increase in concentrations, as measured by EDS, suggests that these elements migrate and form intermetallic compounds or precipitates within specific microstructural regions. Highlighted by the green color are the alloying elements which form the precipitates (Cr, Mo, V).

**Figure 6:**
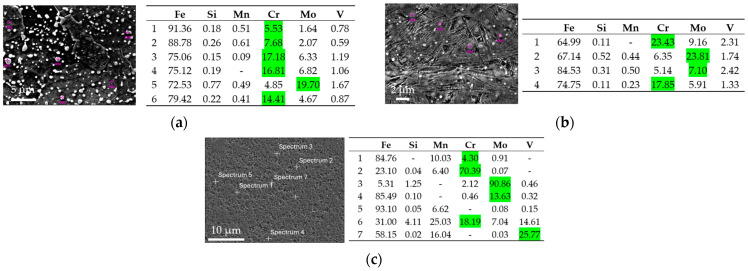
Precipitates of: (**a**) Conv sample, mag. 1000×; (**b**) Conv + RS sample, mag. 6000×; (**c**) SLM + RS sample, mag. 3000×.

The microstructure after soft annealing depicted in Figure 4a consists of a ferritic–pearlitic structure with fine carbides dispersed throughout the structure. The grain size was 10 to 15 µm, as can be deduced from Figure 5a. The carbides were Cr-based and Mo-based, as can be seen in Figure 6a. The BCC phase (ferritic–pearlitic structure) represents 99.8% of volume and the FCC phase (residual austenite) only 0.2%. The hardness of the formed structure was 172 HV1.

After rotary swaging of the Conv state, as shown in Figure 4b, the structure formed was martensitic, with a certain amount of residual austenite. This conclusion was made on the basis of hardness measurements. The hardness of the matrix reached 613 HV1. The grain size was between 3 and 5 µm, as can be seen in Figure 5b. The determined proportion of the BCT (martensite) phase was 98.2%, and that of residual austenite was 1.8% (Figure 5b). A large number of homogeneously dispersed fine Cr-based and Mo-based precipitates were present in the microstructure (Figure 6b).

The microstructure of the as-printed SLM sample in Figure 4c consists of a cellular structure with a cell size of 2 to 5 µm. The hardness of the structure was 560 HV1. Based on the hardness, the structure was assumed to be martensitic. The amount of residual austenite and martensite in the structure was 3.7% and 96.3%, respectively (Figure 5c).

The SLM sample microstructure after rotary swaging is displayed in Figure 4d. It shows a finer microstructure with a grain size of 3 to 4 µm, as can also be seen in Figure 5d. The hardness reached after rotary forging was similar to that of the Conv+RS sample, which was 606 HV1. This indicates that it is a martensitic microstructure. The microstructure will consist of 98.5% martensite and 1.5% residual austenite (Figure 5d). The structure also consists of very fine, homogeneously distributed Cr-, Mo-, and V-based precipitates (Figure 6c).

In the determination of the residual austenite, note that the measurements were carried out on a very small area (200 × 200 µm^2^ grid), and the proportion of residual austenite can be thus misleading. Furthermore, the ratio of the BCC (or BCT) phase to FCC is slightly misleading because the phase analysis does not include the proportion of precipitates in the microstructure.

### 3.2. Neutron Diffraction Results

**Figure 7:**
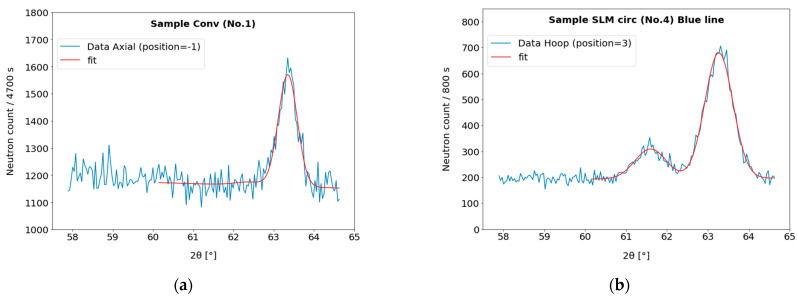
The examples of the measured and fitted diffraction peaks. (**a**) Sample Conv, the axial component; note the small 110 ferritic phase peak/background ratio for this 40.8 mm diameter sample and the necessary long counting time. (**b**) Sample SLM-circular, the hoop component measurement; both residual 111 austenite (**left**) and 110 martensite (**right**) peaks are visible.

Two examples of the as-measured data (for the full set of the measured data see Supplementary File Data S1), one with a relatively high peak intensity and one with low intensity, are shown in Figure 7. For the samples No. 2, 3, 4, and 5, the martensitic peak was observed, in which its individual components of tetragonal structure (110 and 101) cannot be distinguished due to the relatively low carbon content of the used Dievar steel. Therefore, this diffraction maximum (2*θ* angular position of around 63.2°) is treated as one peak and denoted as 110.

The fit to the measured peaks was performed by using the *lmfit*, *leastsq* method (Levenberg–Marquardt). Weighting was performed using statistical errors (reciprocal value of square root of the neutron count).

The peak fitting led to the integral intensities and precise positions of the diffraction angle 2*θ_hkl_* for all the scanned points and all orientations. The peak positions were transferred to the apparent strains using Formulas (2) and (3). Before the calculation of the individual components of the residual elastic stress, a treatment of the so-called pseudostrains, which appear for the scanned points in the vicinity of the sample surface, was carried out. The procedure is described in the next chapter.

## 4. Residual Strain and Stress Evaluation

### 4.1. Pseudostrain and Other Disturbing Effects Treatment

The resulting peak positions and integral intensities were processed by STRESSFIT software, v1.2.0 [83,84] to account for the pseudo-strains caused by the inhomogeneous sampling by neutrons. This inhomogeneous sampling is caused by the following:

- Only partial use of the instrumental gauge volume at the sample boundaries;
- Absorbtion across the gauge volume.

The method employs a list of neutron sampling data generated by the Monte Carlo simulation of the instrument at a given setup [85]. These sampling data are subsequently used in a convolution procedure, which yields simulated “as-measured” intensity and strain distributions, assuming any model distribution of intrinsic scattering intensity, lattice strain, and *d*_0,*hkl*_. This procedure also yields the sampling center of “gravity” (i.e., true information depth), which differs from the nominal scan position, especially at the sample boundaries. Further, the sampling width of the gauge volume, which depends on the position in the sample and decreases significantly when approaching the edges, is determined reliably. Also note that the sampling width is different for the radial, and for the axial and hoop orientations.

The used method enables to determine strains (and consequently stresses) reliably also in the vicinity of the surface.

### 4.2. Determination of the Unconstrained Lattice Spacing

For the determination of strains on an absolute scale, and consequently the correct calculation of stresses using Equation (4), it is necessary to determine the unconstrained value of *d*_0,*hkl*_ of the used reflection. The homogeneous macroscopic distribution of the ferritic/martensitic phase and the retained austenite in the samples was assumed. Then, the *d*_0,*hkl*_ determination was conducted using the stress balance condition in the axial direction [74,86,87] applicable for cylindrical samples.

Note that the stress balance calculation was performed individually for each sample, as it was observed that the amount of the FCC phase varies between the samples. A consequence of the different amount of the FCC phase can be the different composition of the BCC or BCT phase. It could then cause an observable shift of the unconstrained value of *d*_0,110_ for the ferritic/martensitic phase. Performing the stress balance calculation for each sample individually removes this uncertainty.

The uncertainty of *d*_0,110_ was determined by the error propagation of the statistical uncertainties of the measurement through the applied stress balance condition for each individual sample. This systematic uncertainty Δ*d*_0,110_/*d*_0,110_ varies between the samples in the range of 160 ÷ 400 × 10^−6^.

### 4.3. Residual Strains

**Figure 8:**
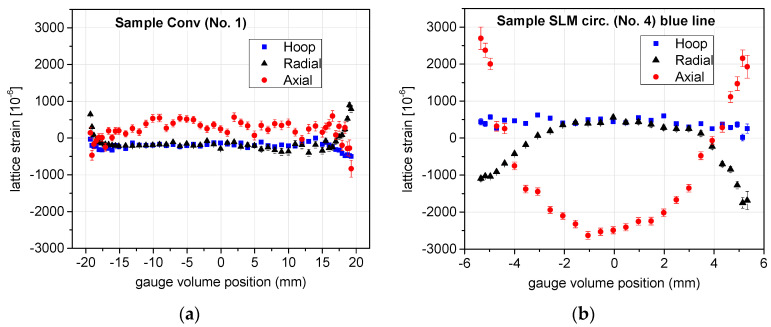
The determined components of the strain after removing the pseudostrain for two of the samples: (**a**) No. 2 (Conv + RS) and (**b**) No. 4 (SLM-circular).

The above treatment led to the components of the residual strain distribution. An example of the output is shown in Figure 8.

The error bars in Figure 8 are given by the statistical errors of the measured peaks propagated to the determined strains according to [68] through the determined angular position of the peak and its uncertainty. The error bars do not include possible systematic errors due to the uncertainty in the determination of the unconstrained value of the lattice spacing *d*_0,110_ for the BCC/BCT phase. The systematic uncertainty of Δ*d*_0,110_/*d*_0,110_ discussed in the previous sub-section would shift the whole strain curve for the given sample vertically, and influence in the similar way also the stress components calculated from the strains.

### 4.4. Residual Stresses

The residual stresses were determined from the above evaluated strains using Formula (3) with the elastic constants *E_hkl_* = 210.5 GPa and *ν_hkl_* = 0.25 taken from [68], valid for the BCC phase. In the case of BCT, the availability of reliable elastic constant values is limited. Nevertheless, a systematic deviation upwards in the determined magnitude of the stress in the range of 10% could be expected [88].

**Figure 9:**
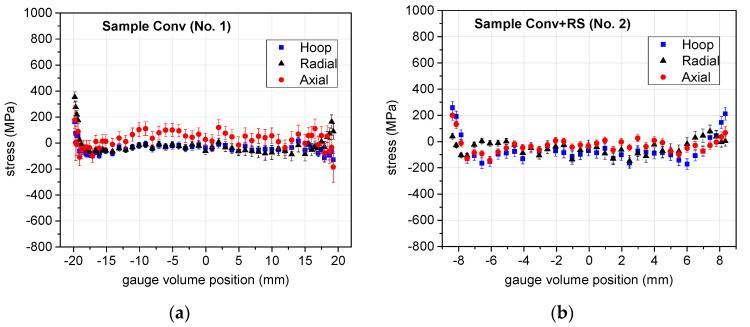
Three components of the stress (points with the error bars) throughout the sample diameter (the gauge volume center of the weight is plotted on the horizontal scale) for the Conv samples: (**a**) stress components along the scanned line in the Conv sample (No. 1), (**b**) stress components along the scanned line in the Conv + RS sample (No. 2). The vertical scale is intentionally selected ranging from −800 MPa to +1000 MPa in order to facilitate comparison with the additively manufactured samples’ results shown in the next figure.

**Figure 10:**
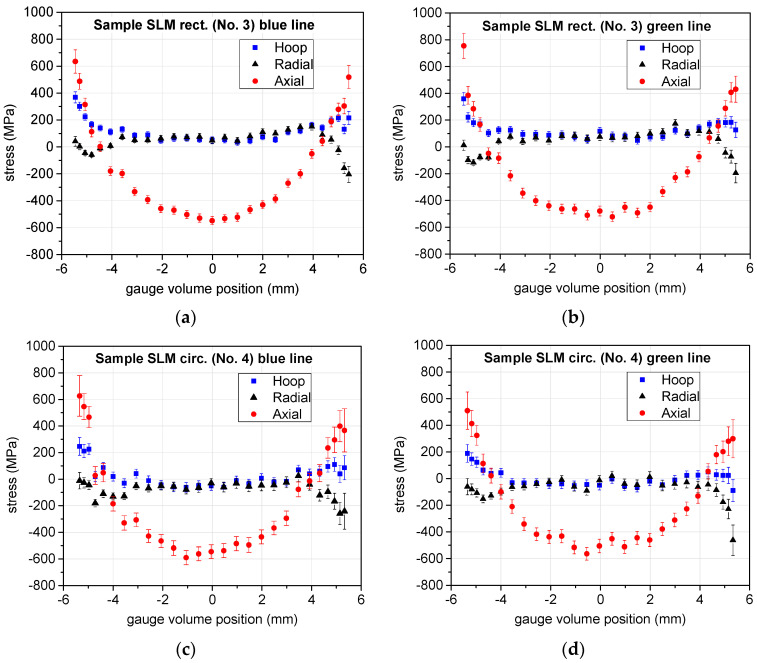

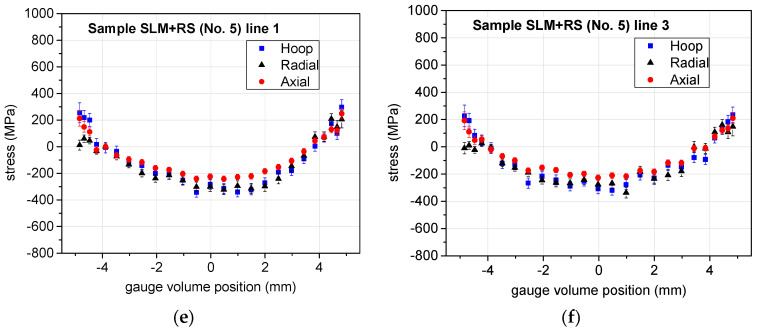
Three components of the stress (points with the error bars) throughout the sample diameter for the AM samples: (**a**,**b**)—stress components along two perpendicular scanned lines for the SLM-rectangular sample (No. 3), (**c**,**d**)—stress components along two perpendicular scanned lines for the SLM-circular sample (No. 4), and (**e**,**f**)—stress components along two perpendicular scanned lines for the SLM + RS sample (No. 5).

Figure 9 and Figure 10 show the resulting residual stress profiles. In the graphs, the negative values characterize compressive residual stresses, while the positive ones characterize residual stresses in tension. The AM samples were scanned along two mutually perpendicular lines (denoted in the figures as the “blue line” and “green line”, or “line 1” and “line 3”); therefore, the resulting stress components are displayed in two separate graphs for these two scan lines. For the Conv sample (i.e., No. 1), Figure 9, the errors are relatively large throughout the sample, as the large sample diameter has the consequence of a very long flight path of the neutron in the material, causing the large attenuation of the beam and—consequently—lower peak intensity.

## 5. Discussion

The Conv sample (diameter 40.8 mm) has a relatively low level of stress for all three components throughout the volume (see Figure 9). In the central region, the hoop and the radial components seem to be slightly compressive, while the axial component is tensile. The situation is rather uncertain near the surface when comparing the values at both ends of the scan line. The conventional sample Conv+RS swaged from 40.8 mm to 18.8 mm has (rather surprisingly) also a relatively low stress level, although a change from nearly zero or tensile (for the axial component) stress to the compressive one in the central part is clearly visible for all the components. In the vicinity of the surface, tensile stresses are visible, mainly for the hoop and axial components.

The samples 3, 4, 5 (SLM-rectangular, SLM-circular, SLM + RS) were measured in two perpendicular directions. Within the experimental errors, the results are the same for the two mutually perpendicular scans (see SLM-rectangular, SLM-cylinder, and SLM+RS in Figure 10). Therefore, we can (with a high degree of probability) state that the cylindrical samples have an axially symmetric distribution of stresses, and the rectangular one has the same distribution in both directions perpendicular to the surfaces of the bar.

The SLM manufacturing alone causes relatively large axial compressive stress (exceeding −500 MPa) in the center, whereas the hoop and radial stresses are nearly zero in the bulk (see SLM-rectangular and SLM-cylinder in Figure 10). Near the surface, the axial stress rises to become relatively large tension stress (larger than 400 MPa) at the edge, whereas the hoop and radial components remain rather moderate also nearby the surface. The range of the measured residual stresses seems to be slightly larger for the rectangular than for the circular SLM sample. Nevertheless, the stress distribution is qualitatively very similar in the measured lines of the SLM-rectangular and SLM-circular samples.

It can be observed that the conventionally (Conv) and AM (SLM)-prepared bars have a completely different distribution of the axial residual stress (see Conv in Figure 9 and SLM-cylinder in Figure 10). The selective laser melting method led to very significant axial stress values with a very pronounced distribution throughout the sample diameter, whereas the Conv sample does not show any such significant pattern.

The rotary swaging of the SLM sample changed very significantly the stress distribution in the SLM bar (see SLM + RS with respect to the SLM-rectangular and SLM-cylinder samples in Figure 10). Although the axial stress absolute value halved both in the central part and near the surface, it remained still relatively large. The absolute value of the other two components, hoop and radial, significantly increased in the central region, but not nearby the surface. The distribution of the stress for the axial, radial, and hoop components is then very similar in the rotary swaged SLM sample, not only qualitatively, but also quantitatively. It indicates the hydrostatic character of the stress. All three components are in compression in the central part of the sample, but in tension in the vicinity of the surface.

Although tension stress is not a favorable factor near the surface, the positive effect of the rotary swaging treatment can be seen. The tensile stress for the axial component very significantly decreased after rotary swaging of the SLM-fabricated bar (the consequence is the lower probability of crack formation) and—moreover—the stress became predominantly hydrostatic, which is the type of stress not causing deformation.

A comparison of samples 2 and 5 shows the significant difference of stress distribution in the material after very similar imposed deformation (see Table 2). The question is whether the difference is caused by the different initial state (Conv × SLM fabrication) or by the original size of the sample, which could cause only the surface layer (i.e., not the central part) to be significantly influenced by rotary swaging for the originally larger diameter sample. As the stress states of the initial samples (Conv and SLM) are different, with the SLM having a significantly larger stress distribution, the difference after rotary swaging is rather ascribed to the difference in the initial state, from which the RS started to redistribute the stresses.

Given the complexity of the residual stress components’ profiles, a simply overviewable indicator of the overall stress state in the material after the rotary swaging processing can be used as well. It is well known that the plastic deformation of metals is stimulated solely by the deviatoric (shape-changing) component of the stress state, often termed the von Mises stress, and is unaffected by the hydrostatic stress component. The following von Mises formula,

(5) $$\sigma_{VM}=\sqrt{\frac{1}{2}[{{(\sigma }_{x}{-\sigma }_{y})}^{2}+{{(\sigma }_{y}{-\sigma }_{z})}^{2}+{{(\sigma }_{z}{-\sigma }_{x})}^{2}\;]},$$

and the hydrostatic stress formula,

(6) $$\sigma_{H}=\frac{\sigma_{x}{+\sigma }_{y}+\sigma_{z}}{3}$$

are used to calculate these parameters. Note that the shear stresses are neglected in these formulas, and the above von Mises relation (5) then only accounts for the normal stress differences (or, equivalently, the deviatoric stress differences).

**Figure 11:**
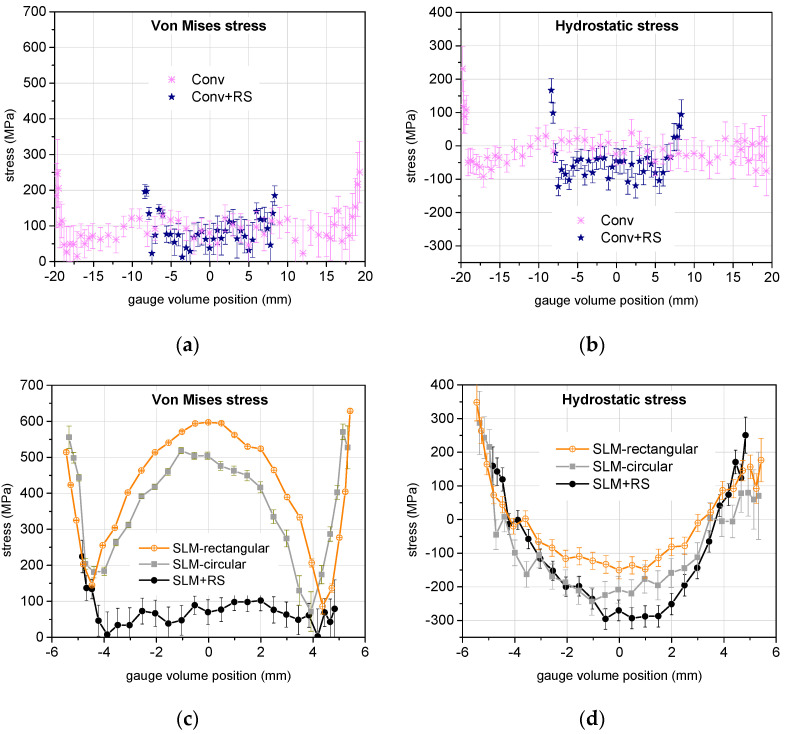
(**a**,**c**) Von Mises stress and (**b**,**d**) hydrostatic stress calculated from the three components of the residual elastic stress. (**a**,**b**) show the stress for the conventionally manufactured bars while (**c**,**d**) show the stress for the additively manufactured bars.

Von Mises and hydrostatic stresses are presented in Figure 11 separately for the conventionally and for the additively manufactured bars. Nevertheless, the stress scales are the same for the von Mises stress and for the hydrostatic stress, respectively, in order to enable easy comparison between the two types of manufacturing.

The graphs show clearly that conventional manufacturing creates a very low or moderate residual stress level, both deviatoric (von Mises) and hydrostatic, whereas the additive manufacturing produces a high level of both deviatoric and hydrostatic stress.

The rotary swaging treatment in conventionally produced material causes no significant change in the deviatoric stresses, but it slightly increases the absolute level of the compressive hydrostatic stress in the bulk of the swaged bar.

The two SLM-manufactured bars (SLM-rectangular and SLM-circular) exhibit qualitatively the very similar character of the residual stresses, both von Mises and hydrostatic. The difference is probably caused by the different geometry of the additive manufacturing. The deviatoric stress for the SLM samples (without swaging) is the largest at the surface and in the center of the bars, and the lowest 1.5 ÷ 2 mm below the surface. These samples have a very similar value of deviatoric stress near the surface, but the SLM-rectangular sample has visibly larger deviatoric stress in the sample center. Also, it should be noted that the residual stress was measured along the lines perpendicular to the surfaces of the rectangular samples. It was not determined along the diagonals of the cross-section, where it could exhibit still larger values of the residual stress or a larger range of stresses within the sample volume. From this consideration, it is not likely that the rectangular cross-section of the SLM-produced bar would be a better initial product for the subsequent rotary swaging than the circular cross-section.

The most significant finding concerns the additively manufactured and rotary swaged bar. (Figure 11c) shows that rotary swaging released nearly fully the deviatoric residual stresses throughout the volume of the bar. Nevertheless, the level of the hydrostatic stress (Figure 11d) remained still high, approximately −300 MPa in compression in the bar center, and its character remained the same as for the SLM bars without swaging: positive in the vicinity of the surface, but negative (i.e., compression) in the central region. Table 3 has been produced to enable an overview and summary of the results. A detailed description of the evaluation of residual stresses has been discussed above.

## 6. Conclusions

The neutron diffraction technique was used for the determination of residual stresses in the Dievar steel for demanding applications after two methods of manufacturing, as well as after rotary swaging post-processing.

The materials were initially evaluated for microstructure, hardness, the proportion of individual structural phases, and precipitates. The manufacturing process for each material has an effect on the extracted microstructure and hardness, i.e., the Conv (ferritic-perlitic; 172 HV1), the Conv+RS (martensitic; 613 HV1), the SLM (martensitic; 560 HV1), and the SLM + RS (martensitic; 606 HV1). The grain size of the Conv state was 10 to 15 µm, the Conv + RS state was 3 to 5 µm, and the SLM + RS state was 3 to 4 µm. The additively manufactured sample had a cellular structure with a cell size of 2 to 5 µm. The Conv sample had a BCC lattice and in the other cases, i.e., Conv + RS, SLM, and SLM+RS, the structure consisted of a BCT lattice. All states showed some amount of residual austenite (FCC phase) with the Conv = 0.2%, the Conv + RS = 1.8%, the SLM = 3.7%, and the SLM + RS = 1.5%. The Conv and the Conv + RS samples contained Cr-based and Mo-based precipitates, whereas the SLM+RS sample contained Cr-based, Mo-based, and V-based precipitates.

The conventionally manufactured sample bar exhibited a low level of all the components of the stress. After swaging, the residual stresses changed only slightly, being lower than 250 MPa with the largest magnitude (in tension) nearby the surface. On the other hand, it was observed that SLM manufacturing causes relatively large axial compressive stress (exceeding −500 MPa in the center), thus exhibiting a significantly different distribution of the axial residual stress than the conventional process. The SLM-caused axial stress is largely tensile near the surface while compressive in the rest of the bar volume.

In contrast to the conventionally prepared material, the rotary swaging of the SLM sample (with very similar imposed strain) changed the stress distribution enormously. The rotary swaging significantly reduced the residual tensile stress in the vicinity of the surface of the SLM+RS bar. It can have a huge effect on crack formation and propagation at the surface. Moreover, the magnitude of the tensile stress near the surface is approximately the same as for the conventionally prepared and rotary swaged material.

The calculation of the deviatoric (von Mises) residual stress confirmed the high level of the residual elastic stress in the SLM-fabricated samples relative to the stresses in the conventional sample. Nevertheless, it also showed the stress release through the rotary swaging in nearly the whole volume of the sample. This finding underlines the positive effect of rotary swaging on the residual stresses in SLM-manufactured components.

No advantage of the rectangular shape of the SLM-manufactured sample (when compared to the circular cross-section bar) for rotary swaging treatment can be deduced from the measured data.

These findings demonstrate the potential of rotary swaging as a post-processing technique to optimize the mechanical properties and residual stress profiles in tool steels, particularly those produced by selective laser melting. The reduction in the residual stress level, particularly the reduction in the tensile stresses near the surface, significantly enhances the material’s resistance to crack initiation and propagation, which is crucial for components subjected to cyclic loading and high thermal stresses in industries such as aerospace and automotive.

Moreover, the study highlights the importance of further investigating how different printing strategies (geometry, orientation) in SLM influence residual stress distribution and how these can be optimized through mechanical post-processing. This would provide critical insights for tailoring manufacturing processes to meet specific application requirements, paving the way for the broader adoption of additive manufacturing in high-performance industrial applications.

## Figures and Tables

**Table 1:** Chemical composition of Dievar alloy (wt. %).

C	Si	Mn	Cr	Mo	V
0.35	0.20	0.50	5.00	2.50	0.60

**Table 2:** Overview of the investigated samples.

Sample Nr.	Sample Name	Cross-Sectional Dimension	Diameter after Reduction by RS	Imposed Strain (-)
1	Conv	ø 40.8 mm	N/A	0
2	Conv + RS	ø 40.8 mm	ø 18.8 mm	1.55
3	SLM-rectangular	12 × 12 mm^2^	N/A	0
4	SLM-circular	ø 12 mm	N/A	0
5	SLM+RS	ø 25.4 mm	ø 11.1 mm	1.65

N/A denotes Not Applicable.

**Table 3:** Overview of the investigation results for various fabrications and processing of Dievar.

	Conv	Conv + RS	SLM	SLM + RS
Microstructure	Ferritic-perlitic	Martensitic	Martensitic	Martensitic
Grain size (µm)	10–15	3–5	2–5	3–4
Hardness (HV1)	172	613	560	606
BC(C,T)/FCC	99.8/0.2	98.2/1.8	96.3/3.7	98.5/1.5
Carbides-based	Cr, Mo	Cr, Mo	-	Cr, Mo, V
von Mises stress	Low level, minimal fluctuations	Lower residual stress, more uniform	Significant, pronounced maxima at surface and in center	Overall low level, reduced surface value
Hydrostatic stress	Very low level, uniform—except surface	Low level, uniform—except surface	Parabolic-like distribution, tensile near the surface	Parabolic-like distribution, tensile near the surface
Overall characteristics	Low residual stress and uniform distribution	High hardness, improved strength of the material	High hardness and residual stress on the surface, risk of surface cracking	Improved residual stress level
Potential applications	Suitable for less demanding applications	Demanding applications	Demanding application with certain limitations	Suitable for more demanding applications (fatigue resistance)

## Data Availability

The original data supporting the research are contained within the article and its Supplementary Materials.

## Supplementary Materials

The following supporting information can be downloaded at: https://www.mdpi.com/article/10.3390/ma17235706/s1, Supplementary File S1: Raw data from the neutron diffraction measurement.
